# Blood lead levels in children and soil lead contamination in a former mining area in Germany

**DOI:** 10.1097/EE9.0000000000000459

**Published:** 2026-02-09

**Authors:** Lea John, Katja Radon, Walter Schmotz, Finn Sonnemann, Michael Hoopmann, Nathalie Costa Pinheiro, Martin Hepp, Stefan Rakete, Dennis Nowak, Laura Wengenroth

**Affiliations:** a Institute and Clinic for Occupational, Social and Environmental Medicine, LMU University Hospital, LMU Munich, Munich, Germany; b District of Goslar, Department of Construction & Environment – Soil Protection and Waste Monitoring, Goslar, Germany; c Public Health Agency of Lower Saxony, NLGA, Hanover, Germany; d District of Goslar, Department of Public Health Services, Goslar, Germany

**Keywords:** Lead exposure, Blood lead level (BLL), Mining, Environmental epidemiology, Children, Germany

## Abstract

**Background:**

Lead-contaminated soils in former mining areas can pose an environmental hazard for decades. In the former mining district of Goslar, Germany, where mining ceased in 1988, soil lead exposure remains a public health concern, with median soil lead concentrations reaching up to 1,500 mg/kg in residential areas. Children are particularly vulnerable to lead exposure due to physiological and behavioral factors. This study aimed to assess blood lead levels (BLLs) in children and identify key exposure pathways.

**Methods:**

In a cross-sectional study conducted in 2023/24, BLLs were measured in 310 5–7-year-old children. Guardians provided information on potential exposure pathways, including handwashing behavior, hand-to-mouth contact, consumption of homegrown/foraged food, outdoor activity, and secondhand smoke. Children’s locations of residence and recreation were assessed, and soil contamination data were available. Factors were analyzed using bivariate comparisons and adjusted linear regression model.

**Results:**

The BLL geometric mean was 22.7 μg/L. Of the children, 51% exceeded the German reference values (19/22 μg/L girls/boys), and 24% exceeded the US reference value (35 μg/L). Thirteen percent had BLLs above 50 μg/L, the World Health Organization benchmark for preventive action. Soil contamination emerged as the main exposure pathway. Children living in very highly contaminated areas had BLLs 29% higher (95% CI: 7%, 56%) than those in the least contaminated areas.

**Conclusions:**

These results emphasize the importance of continuing and strengthening preventive measures to reduce lead exposure and intake. Given similar historical contamination in other regions, more extensive environmental assessments and targeted interventions are required to protect children’s health in former mining areas worldwide.

**What this study adds**

Decades after mining ceased in the district of Goslar, Germany, children still exhibit elevated blood lead levels, with over half exceeding national reference values and 13% above World Health Organization preventive benchmark. The study demonstrates that soil lead contamination remains the dominant exposure pathway and quantifies its contribution to children’s blood lead levels. By combining environmental measurements with detailed exposure information, this research provides robust evidence on the impact of mining related soil contamination on children’s blood lead level, contributing valuable data for exposure assessment and epidemiological understanding in former mining areas worldwide.

## Introduction

Lead is a persistent and toxic metal that has been extensively used in various industrial and consumer products.^1^ As a result of its widespread use, lead can be found in air, water, dust, and soil.^2^ Lead contamination in soil of former mining and smelting areas can remain for decades after industrial activities have ended.^3–8^

Despite global reductions in environmental lead exposure – primarily due to bans on leaded gasoline^7,9–12^ – lead contamination continues to pose a widespread environmental challenge.^13^ In 2021, lead exposure was estimated to be responsible for approximately 1.5 million human deaths worldwide,^14^ and the global economic costs attributable to lead exposure in humans were estimated at US$6.0 trillion in 2019, largely driven by ongoing exposure in low- and middle-income countries.^15^

According to current research, there is no lead concentration in the body that can be considered as harmless. Children are especially susceptible to the harmful effects of lead. Even blood lead levels (BLLs) starting at 50 µg/L (micrograms per liter) can impair the development of children’s central nervous system.^16–20^ In its 2010 risk assessment, the European Food Safety Authority set benchmark dose lower confidence limits for critical health outcomes. The benchmark dose lower confidence limits for developmental neurotoxicity was 12 µg/L, equivalent to an estimated daily intake of 0.50 µg/kg body weight, and was associated with a one-point reduction in intelligence quotient.^21^ The World Health Organization (WHO) recommends initiating preventive measures when BLLs reach 50 µg/L. At levels exceeding 450 µg/L, or when clinical symptoms of lead toxicity are present, the WHO further advises individual medical treatment, such as chelation therapy.^17^ Young children are at heightened risk due to frequent hand-to-mouth contact, mouthing of contaminated objects, and playing close to or on the ground, where they may come into contact with lead-contaminated soil or dust.^17^ Adults absorb 10%–15% of the ingested lead, while children absorb up to 40%–50%, making ingestion a particularly critical pathway of lead exposure.^22,23^ In addition to environmental sources, further exposure pathways may include secondhand tobacco smoke,^24^ contact with lead-containing ammunition (e.g., via recreational shooting),^25^ and the consumption of contaminated food products.^26,27^ Consequently, children can be at risk of lead exposure, without living in areas with significant soil lead contamination. This is reflected in the population-based German Environmental Survey (GerES V), which reported a geometric mean (GM) BLL of 9.5 µg/L among children aged 3–17 years.^28^

### Reference values for lead

In the absence of a safe threshold or a human biomonitoring value for lead exposure, population-based reference values (RVs) for BLLs are used. As statistical constructs, these RVs do not indicate health-based thresholds but reflect background exposure levels in the general population. In Germany, RVs for BLLs are age- and sex-specific and based on the 95th percentile of the general population; thus, exceedances are statistically expected in 5% of individuals. The German Human Biomonitoring Commission published updated RVs in 2025, derived from data collected during GerES V conducted between 2014 and 2017. For children aged 3−10 years, the RVs are 19 µg/L for girls and 22 µg/L for boys.^28,29^ In 2021, the Centers for Disease Control and Prevention in the United States set an RV of 35 µg/L for BLLs in children. This RV is based on the 97.5th percentile of BLLs measured in US children 1−5 years of age who participated in the National Health and Nutrition Examination Survey during the 2015–2016 and 2017–2018 cycles; thus, exceedances are statistically expected in 2.5% of individuals.^30,31^

### Lead-contaminated soil in the district of Goslar

**Figure 1.**
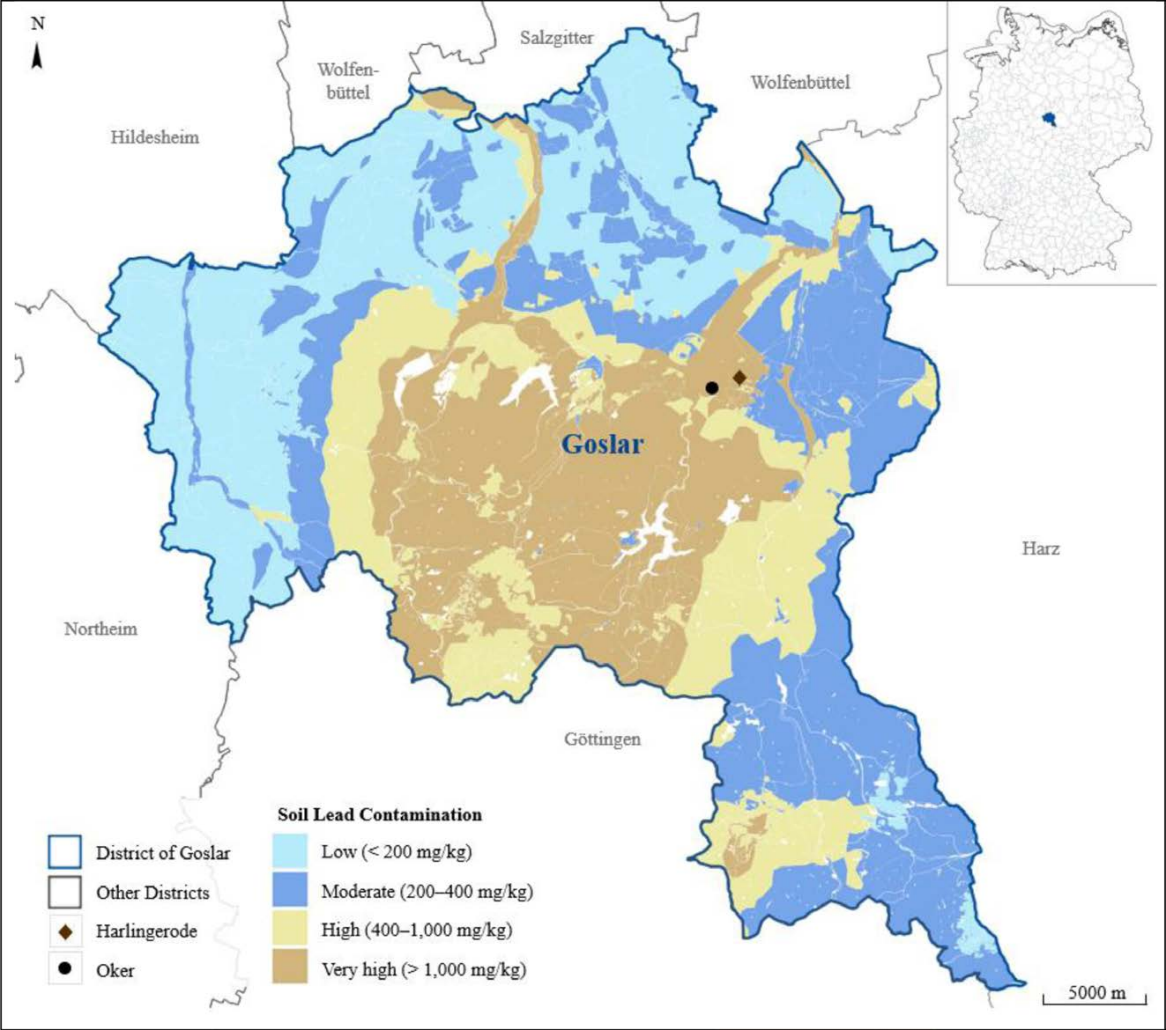
Map of the district of Goslar including Oker and Harlingerode with soil lead contamination. White areas in the Goslar district indicate water bodies. The figure was created using QGIS (version 3.34.6) based on data from the Department of Construction & Environment – Soil Protection and Waste Monitoring in Goslar,^32^ the Lower Saxony State Office for Geoinformation and State Survey,^33^ and the Federal Agency for Cartography and Geodesy.^34^ Details on soil measurements in residential areas can be found in Supplementary Table A1; https://links.lww.com/EE/A408.

In the district of Goslar (Lower Saxony, Germany; Figure 1), the mining and processing of ores containing lead, zinc, copper, and silver, among other metals ceased in 1988.^35^ Soil samples collected since 1998 revealed severe metal contamination, with lead concentrations reaching up to 30,000 mg/kg^36^ – far exceeding the German maximum permissible lead concentration in soil for residential areas (400 mg/kg) and playgrounds (200 mg/kg), which were established based on human health risk assessments.^37^ The regions of Oker and Harlingerode (Figure 1, marked with a black dot and a brown rhombus, respectively), in particular, were identified as historical contamination hotspots due to intensive mining activities and large-scale metal production.^35^

A cross-sectional study conducted in 1980 revealed that children 6−12 years of age living in Oker (Figure 1, black dot) had a median BLL of 228 µg/L.^38^ By comparison, a study carried out in 1992/93 in the former mining area of Hettstedt, located approximately 70 km from the district of Goslar, found lower values in children 5−14 years of age, with a GM of 38 µg/L.^39^ In response to the elevated BLLs in Oker, local authorities in Goslar implemented a few remediation measures over the following decades. However, these efforts were limited to areas with lead soil contaminations above 1,000 mg/kg (Figure 1, areas shaded in light brown).^40,41^

Decades later, in 2021, a cross-sectional study was again conducted among 6−10 year-old children in the highly contaminated areas Oker (Figure 1, black dot) and Harlingerode (Figure 1, brown rhombus). Results showed that although BLLs had declined to 17 µg/L (both median and GM), 48% of the children still exceeded the German RVs (5% expected^29^), and 8% exceeded the US RV (2.5% expected^31^).^42^ In this study, (almost) no exposure pathways were conducted.

The aim of the present study was therefore to assess BLLs in preschool children across the whole district of Goslar – not only Oker and Harlingerode – and to identify exposure pathways contributing to elevated BLLs, to inform and further develop targeted, evidence-based public health interventions.

## Methodology

### Study design

The cross-sectional study was conducted between September 2023 and June 2024 in the district of Goslar, Lower Saxony, Germany. Along with the notification for the school entry health check for the 2023/24 academic year, guardians received an invitation to the study. Participants received information materials, an informed consent form, and a short questionnaire. They were invited to participate in a face-to-face interview and to have their children’s blood sampled to measure the BLLs. Reminders were sent 2 weeks before the individual appointment for the school entry health check. Individual blood lead results were sent to guardians via letters, and individual environmental medical counseling sessions were offered. The study was approved by the Ethics Committee at the Medical Faculty of Ludwig Maximilian University of Munich (approval no. 23-0178). Participation in the study was voluntary. Written informed consent was obtained from the legal guardians of all participants.

### Study population

All children 5−7 years of age in the district of Goslar (N = 1,196) were invited to participate in the study. Of these, 357 children (30%) took part. A total of 310 children (26%) with complete data were included in the final analyses.

### Study instruments and variable definitions

The written questionnaire covered sociodemographic characteristics, including the child’s sex and age, as well as the guardian’s education and professional qualifications. It also assessed the child’s exposure to secondhand tobacco smoke and ammunition.^43,44^ In addition, structured face-to-face interviews with guardians were conducted by trained interviewers. They covered hygiene behavior (handwashing and hand-to-mouth contact), consumption of homegrown and foraged food, location of residence, location of kindergarten (preschool childcare), and outdoor activities (location and time spent in gardens – private and those of friends/relatives – playgrounds or sports fields, forests or agricultural areas, and waterside locations).^45,46^ Variable definitions are shown in Table 1. All questions were derived from validated questionnaires.^43–46^

**Table 1.** Variable definitions

Variable name	Variable description	Categories^a^	Category definition
Sex	Child’s sex	Female, male, diverse	─
Age	Child’s age	5 years, 6 – 7 years	─
Socioeconomic status	Based on the guardian’s education and professional qualifications	Low, moderate, high	Low: all other Moderate: ≥1 guardian with intermediate school degree/vocational training High: ≥1 guardian with a university degree
Secondhand tobacco smoke	Exposure to tobacco smoke from household members	No, yes	No: never Yes: less than once a week, once a week, more than once a week, or daily
Ammunition	Exposure to ammunition-related contamination (e.g., via recreational shooting)	No, yes	No: never Yes: seldom, sometimes, often, or always
Handwashing behavior	Handwashing after outdoor play and before eating	Yes, no	Yes: sometimes, often, or always No: never, or seldom
Hand-to-mouth contact	Frequent hand-to-mouth contact	No, yes	No: never, or seldom Yes: sometimes, often, or always
Consumption of homegrown food	Vegetables, fruits, or herbs from gardens	No, yes	No: never, or less than once a week Yes: once a week, more than once a week, or daily
Consumption of food from the forest	Foraged food from forests, such as mushrooms, wild garlic, blueberries, or similar	No, yes	No: never, or less than once a week Yes: once a week, more than once a week, or daily
Locations	Regularly visited places (past 12 months): residence, kindergarten (preschool childcare), gardens (private and those of friends/relatives), playgrounds or sports fields, forests or agricultural areas, and waterside locations	Addresses or coordinates	Linked with soil lead contamination
Soil lead contamination	Lead concentrations in soil at each individual location^b^	Low, moderate, high, very high	Low: < 200 mg/kg Moderate: 200–400 mg/kg High: 400–1,000 mg/kg Very high: > 1,000 mg/kg
Time spent outdoors	Time spent at outdoor locations^c^ (weekly average, past 12 months)	Little, much	Little: ≤ 15 h/week Much: > 15 h/week
Season of blood sampling	Season of blood lead level measurement	Winter, autumn, spring, summer	Winter: December to February Autumn: September to November Spring: March and April Summer: May and June^d^

a Reference category named first.

b Locations = residence, kindergarten (preschool childcare), gardens (private and those of friends/relatives), playgrounds or sports fields, forests or agricultural areas, and waterside locations; data on soil lead contamination provided by the Department of Construction & Environment – Soil Protection and Waste Monitoring in Goslar,^32^ see Supplementary Table A1; https://links.lww.com/EE/A408.

c Outdoor locations = gardens (private and those of friends/relatives), playgrounds or sports fields, forests or agricultural areas, and waterside locations.

d No measurements were taken in July and August.

All study materials were provided in German, and interpreters were available upon request. The instruments were pilot-tested during the first week of data collection.

### Data on soil lead contamination in the study area

Soil lead contamination data were collected between 1998 and 2005 by the Department of Construction & Environment – Soil Protection and Waste Monitoring in Goslar.^32^ Based on these measurements, different contamination areas were specified in the regulation on the “Harz Land Use Planning Area in the District of Goslar” (In German: Verordnung des Bodenplanungsgebietes Harz im Landkreis Goslar), ranging from low (<200 mg/kg) to very high (>1,000 mg/kg) soil lead contamination for residential areas.^36^ The four defined contamination areas (low, moderate, high, and very high) were used as a reference for this study (see 'soil lead contamination' in Table 1). Details of the soil measurements in residential areas can be found in Supplementary Table A1; https://links.lww.com/EE/A408. QGIS (Geographic Information System Installation Guide, QGIS Association, QGIS 3.34.6, Boston, MA) was used to link each child’s location to the respective contamination area.

### Blood sample collection and laboratory analyses

Capillary blood samples were collected from the children’s fingertips after the protocol provided by the US Centers for Disease Control and Prevention.^47^ Lead concentrations were measured using an inductively coupled plasma tandem mass spectrometer (ICP-MS/MS; 8900, Agilent Technologies, Waldbronn, Germany, funded by the German Research Foundation [In German: Deutsche Forschungsgemeinschaft] – INST 86/2091-1), equipped with an I-AS autosampler and an Ultra High Matrix Introduction interface.

The instrument was tuned daily to optimize sensitivity, the oxide ratio, and the doubly charged ion ratio. Samples were analyzed in HMI mode with a four-fold dilution and run in single quadrupole mode using helium as a collision gas to minimize polyatomic interferences. Lead isotopes (^206^Pb + ^207^Pb + ^208^Pb) were quantified using an external calibration. ^159^Tb was used as the internal standard.

All reagents used were of the highest purity grade. Ultrapure nitric acid (Optima, Fisher Scientific, Hampton, NH) and ultrapure water (18.2 MΩ·cm; Direct-Q, Merck, Darmstadt, Germany) were used. Calibration standards were obtained from Agilent Technologies, and lyophilized blood control materials were purchased from Recipe (Munich, Germany, ClinChek 8840, lots 1299 [37.6 µg/L] and 2193 [34.0 µg/L]) and Sero (Billingstad, Norway, Seronorm 210105, lot 2011920 [10.3 µg/L]). Samples were thawed to room temperature on a roll mixer and diluted 1:20 with 0.5% (v/v) nitric acid containing 10 μg/L ^159^Tb.

The limit of quantification(for lead in blood was 0.33 μg/L. Where feasible, each sample was measured twice to ensure reliability and repeatability, and the relative standard deviation (RSD) was calculated. Field blanks were tested in between. Quality control samples were analyzed at the beginning and end of each analytical run and after every 20 samples. Acceptable recovery was defined as 80%–120%. The laboratory also successfully participated in the German External Quality Assessment Scheme.

In this study, background levels in the used blood collection tubes were below the limit of quantification. However, in 8 out of 35 tested field blanks, quantifiable amounts of Pb were measured (<0.72 µg/L equivalent in blood).

### Statistical analyses

All statistical analyses were conducted using R (Posit, PBC, R 4.1.1, Boston, MA). The distribution of BLLs was described using the 25th, 50th (median), 75th, 95th, and 98th percentiles, as well as the arithmetic mean and GM. Additionally, the proportion of children exceeding RVs for BLLs was calculated using the RVs that were in effect in 2025: German RVs of 19 µg/L for girls and 22 µg/L for boys,^29^ US RV of 35 µg/L for both sexes,^30^ and 50 µg/L as the WHO benchmark.^17^

Group comparisons for the continuous outcome variable BLLs were conducted using the Mann–Whitney *U* test for comparisons between two groups, and the Kruskal–Wallis test when comparing more than two groups. Due to the skewed distribution of BLLs, values were log-transformed before linear regression analyses. All regression models were adjusted for sex, age, exposure to secondhand tobacco smoke, handwashing behavior, and the season of blood sampling.

To assess the robustness of our findings, several sensitivity analyses were conducted.

## Results

### Description and blood lead levels of the study population

Overall, BLLs ranged from 4.7 to 206.6 µg/L with a GM of 22.7 µg/L and a 95th percentile of 79.5 µg/L. BLLs of more than half of the children (51%) exceeded the German RVs, and 24% exceeded the US RV (Table 2). Thirteen percent of the children had BLLs above 50 µg/L (WHO benchmark).

**Table 2.** Description and blood lead levels of the study population

	N	Percentile					Mean^a^		Reference value^b^	
		25	50	75	95	98	AM	GM	Germany	United States
		μg/L							N (%)	
Total	310	14.6	20.4	34.2	79.5	130.5	29.7	22.7	157 (50.6)	75 (24.2)
Sex^e^ (***P* < 0.01**)^c^										
Female	136	14.3	17.7	26.6	57.5	68.5	23.5	19.2	58 (42.6)	19 (14.0)
Male	174	15.9	24.1	40.7	93.6	143.4	34.5	25.9	99 (56.9)	56 (32.2)
Age (*P* = 0.08)^c^										
5 years	201	14.4	19.6	31.0	77.3	104.5	27.6	21.6	94 (46.8)	41 (20.4)
6−7 years	109	16.0	23.2	40.2	84.4	153.9	33.4	24.9	63 (57.8)	34 (31.2)
Socioeconomic status^h^ (*P* = 0.46)^d^										
Low	20	11.9	15.8	31.3	72.2	72.3	25.8	19.9	7 (35.0)	4 (20.0)
Moderate	141	15.1	20.6	36.3	63.5	91.6	29.1	23.0	74 (52.5)	37 (26.2)
High	139	14.4	20.4	30.4	83.8	137.7	29.9	22.4	69 (49.6)	30 (21.6)
Secondhand tobacco smoke^i^ (*P* = 0.09)^c^										
No	234	14.4	19.8	31.1	73.2	114.8	28.2	21.8	114 (48.7)	49 (20.9)
Yes	73	15.5	24.7	43.2	91.6	126.8	34.2	25.6	41 (56.2)	24 (32.9)
Hand-to-mouth contact^j^ (*P* = 0.61)^c^										
No	210	14.4	19.5	35.2	87.5	138.9	30.9	22.7	104 (49.5)	54 (25.7)
Yes	95	15.2	22.2	33.3	54.7	74.6	27.2	22.8	51 (53.7)	20 (21.1)
Handwashing behavior^i^ (***P* = 0.04**)^c^										
Yes	242	14.4	19.6	32.0	74.6	114.6	28.0	21.8	115 (47.5)	53 (21.9)
No	65	16.3	26.7	41.8	84.3	154.8	35.7	26.5	40 (61.5)	21 (32.3)
Consumption of homegrown food^k^ (*P* = 0.16)^c^										
No	126	12.5	18.4	38.0	72.4	91.8	28.3	21.2	56 (44.4)	34 (27.0)
Yes	182	15.9	22.0	33.1	82.5	142.3	30.7	23.9	100 (54.9)	41 (22.5)
Consumption of food from the forest^k^ (*P* = 0.10)^c^										
No	279	14.4	20.0	33.6	75.0	121.1	29.0	22.2	139 (49.8)	65 (23.3)
Yes	29	17.3	23.2	40.7	95.8	139.0	36.6	28.3	17 (58.6)	10 (34.5)
Time spent outside^f^ (*P* = 0.24)^c^										
Little	237	14.3	19.7	34.2	86.6	136.3	29.6	22.3	117 (49.4)	57 (24.1)
Much	73	16.2	22.2	34.0	73.3	80.4	29.9	24.3	40 (54.8)	18 (24.7)
Season of blood sampling (***P* < 0.01**)^d^										
Winter	97	11.5	16.2	24.0	55.0	67.2	22.0	17.2	34 (35.1)	11 (11.3)
Autumn	81	16.3	20.4	31.1	59.5	80.8	27.5	22.8	46 (56.8)	15 (18.5)
Spring	79	14.9	22.9	40.5	89.1	117.9	32.9	25.6	44 (55.7)	27 (34.2)
Summer	53	17.7	27.5	51.9	121.8	182.2	42.2	31.3	33 (62.3)	22 (41.5)
Soil lead contamination^g^ at residence (***P* = 0.04**)^d^										
Low	85	14.4	19.5	27.2	53.8	118.1	26.3	21.0	39 (45.9)	13 (15.3)
Moderate	91	14.4	18.3	29.3	55.8	81.6	24.3	20.2	39 (42.9)	14 (15.4)
High	39	15.0	21.3	35.6	61.1	105.6	29.3	23.7	22 (56.4)	10 (25.6)
Very high	95	15.1	27.2	47.5	89.3	178.8	38.0	26.9	57 (60.0)	38 (40.0)

a AM = Arithmetic mean, GM = Geometric mean.

b German reference values: 19 μg/L for girls, 22 μg/L for boys; US reference value: 35 μg/L for both sexes.

c *P* values for continuous BLLs; Mann–Whitney *U* test; statistically significant values (*P* < 0.05) in bold.

d *P* values for continuous BLLs; Kruskal–Wallis test; statistically significant values (*P* < 0.05) in bold; *P* values of pairwise comparisons in Supplementary Table A2; https://links.lww.com/EE/A408.

e No indication of 'diverse'.

f Little: ≤ 15 h/week, much: > 15 h/week.

g Lead in soil (mg/kg): low < 200 mg/kg, moderate = 200−400 mg/kg, high = 400−1,000 mg/kg, very high > 1,000 mg/kg.

h N = 10 missings.

i N = 3 missings.

j N = 5 missings.

k N = 2 missings.

The study population consisted of more boys than girls (56% vs. 44%), with a mean age of 5.4 years (standard deviation 0.5; range 5–7 years). Boys and older children showed statistically significant higher BLLs. Most children came from families with a high socioeconomic status (SES; 46%), while only 7% were from families with low SES. SES showed no association with BLLs.

**Figure 2.**
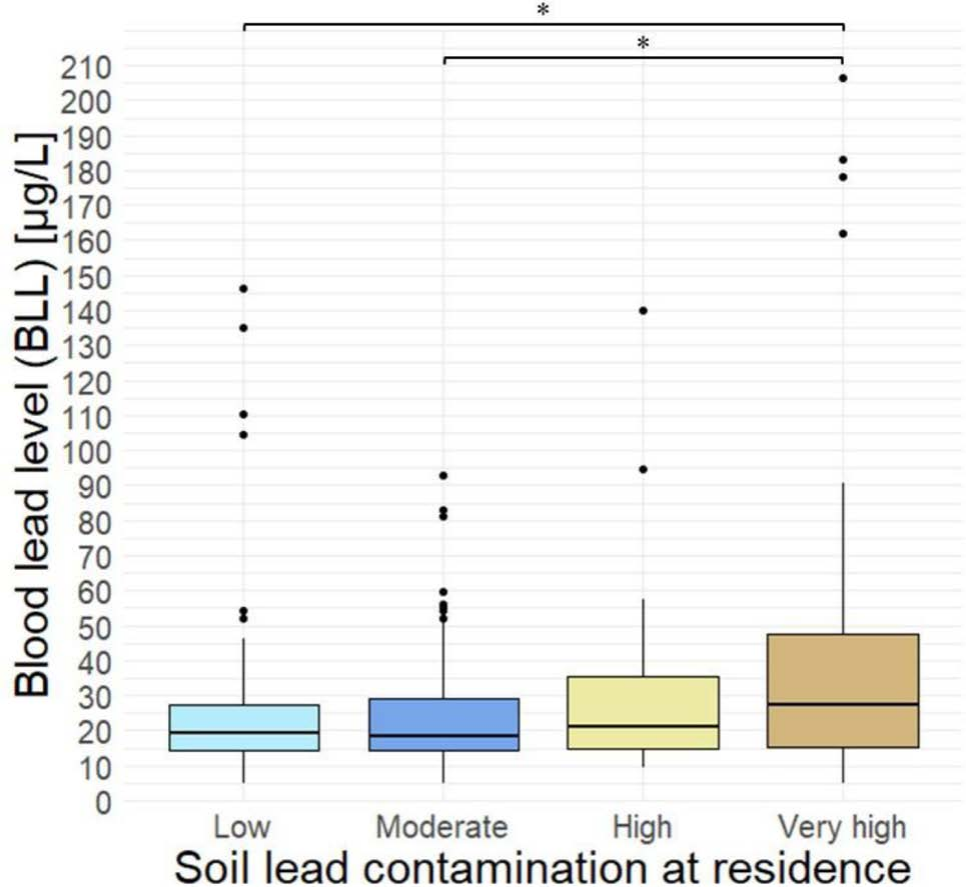
Blood lead levels by soil lead contamination at the residence. *Indicate *P* values < 0.05, others not significant; Lead in soil (mg/kg): low < 200 mg/kg, moderate = 200−400 mg/kg, high = 400−1,000 mg/kg, very high > 1,000 mg/kg.

As shown in Table 2, regular handwashing behavior was associated with statistically significant lower BLLs. Regarding seasonality, BLLs were highest in summer and lowest in winter. Exposure to secondhand tobacco smoke was linked to higher BLLs, although this association did not reach statistical significance (*P* > 0.05). Children living in areas with very high soil lead contamination had statistically significant higher BLLs than those living in low (GM 26.9 vs. 21.0 μg/L, *P* = 0.02) and moderate (GM 26.9 vs. 20.2 μg/L, *P* = 0.01) contaminated areas (Figure 2 and Supplementary Table A2; https://links.lww.com/EE/A408). In these very highly contaminated areas, 60% of children exceeded the German RVs and 40% exceeding the US RV. Elevated BLLs were observed across all soil contamination levels. Even in areas with comparatively lower soil lead concentrations, 46% of children exceeded the German RVs and 15% exceeded the US RV (Table 2).

Further, no statistically significant associations were observed between BLLs and hand-to-mouth contact, the consumption of homegrown food, the consumption of food from the forest, or the amount of time spent outside. An analysis stratified by soil lead contamination areas at residence also showed no statistically significant associations (Supplementary Figure A1; https://links.lww.com/EE/A408). Potential exposure to ammunition was reported by only 7 guardians; due to the small number of cases, this variable was not analyzed further.

### Factors potentially associated with elevated blood lead levels

The adjusted model (Table 3) showed similar results to the bivariate analysis (Table 2). When accounting for potential confounders in the adjusted model, BLLs were 29% higher (95% CI: 7%, 56%) for children living in the highest lead contamination areas compared with children living in areas with the lowest soil lead contamination. To avoid multicollinearity, soil lead contamination at locations other than the residence was not included in the regression model, as the level of lead contamination across locations was strongly correlated.

**Table 3.** Unadjusted and adjusted regression models with blood lead levels as a continuous outcome and soil lead contamination at residence as predictor (N = 304)

	Unadjusted		Adjusted	
	Coefficient (β)	95% CI	Coefficient (β)	95% CI
Intercept	21.04	18.16–24.37	10.60	8.42–13.34
Soil lead contamination at residence				
Low	1	─	1	─
Moderate	0.96	0.78–1.18	1.00	0.83–1.21
High	1.13	0.87–1.46	1.28	1.00–1.63
Very high	**1.25**	**1.02–1.54**	**1.29**	**1.07–1.56**
Sex				
Female	─	─	1	─
Male	─	─	**1.39**	**1.20–1.61**
Age				
5 years	─	─	1	─
6−7 years	─	─	1.16	0.96–1.40
Secondhand tobacco smoke				
No	─	─	1	─
Yes	─	─	1.15	0.97–1.35
Handwashing behavior				
Yes	─	─	1	─
No	─	─	**1.20**	**1.01–1.42**
Season of blood sampling				
Winter	─	─	1	─
Autumn	─	─	**1.47**	**1.20–1.81**
Spring	─	─	**1.70**	**1.38–2.08**
Summer	─	─	**1.80**	**1.44–2.25**

Coefficient β = Exponentiated regression coefficient (β); BLLs were log-transformed; 95% CI = 95% confidence interval; statistically significant values in bold; adjusted R^2^ for adjusted model = 0.19.

### Blood lead levels in relation to soil lead contamination areas

The association between very highly contaminated soil areas and elevated BLLs was not limited to residential addresses. Higher BLLs were also found in children who regularly visited kindergartens (preschool childcare), private gardens, playgrounds or sports fields, as well as forests or agricultural areas located in very highly contaminated areas. In contrast, no associations were found for visits to very highly contaminated friends’ or relatives’ gardens or to waterside locations. These findings are presented in Supplementary Table A3; https://links.lww.com/EE/A408.

### Results of the sensitivity analyses

All sensitivity analyses showed consistent results, supporting the robustness of our findings. Group comparisons with BLL as a binary variable (RV as threshold) revealed similar associations (Supplementary Table B1; https://links.lww.com/EE/A408). The logistic regression analysis on soil contamination at residence confirmed the same pattern of associations (Supplementary Table B2; https://links.lww.com/EE/A408). Also, excluding children with reported exposure to ammunition and those with an RSD greater than 20% between duplicate blood measurements did not substantially change the results of the linear regression model on soil contamination at residence (Supplementary Table B3; https://links.lww.com/EE/A408).

## Discussion

The present cross-sectional study revealed a higher burden of lead exposure in 310 children from the former mining district of Goslar compared with the general population. Overall, half of the children exceeded the German RVs (5% expected), and every fourth child exceeded the US RV (2.5% expected). Furthermore, 13% of the participants had BLLs above 50 µg/L, the WHO benchmark for preventive action. Soil lead contamination was identified as the primary exposure pathway of elevated BLLs.

### Comparison with other studies

In addition to nationwide preventive measures such as the ban on gasoline, the authorities of Goslar implemented local interventions since 2001, including educational programs (e.g., information materials) and the covering and sealing of a few selected contaminated areas.^40,41^ Nevertheless, elevated BLLs were still observed in children across all areas with varying levels of soil lead contamination in the present study. Soil lead contamination has likewise been identified as a major exposure pathway in other former mining regions and reductions in soil lead concentrations have been shown to substantially lower BLLs. For example, a study in Montana, United States, reported a reduction in mean BLLs among children, from 35 to 15 µg/L between 2003 and 2010, following soil remediation.^7^ Similarly, a study from Bangladesh in 2017/18 showed that decreasing median soil lead concentrations from 1,400 mg/kg to 55 mg/kg led to a 35% reduction in children’s median BLLs (from 226 to 148 µg/L), in combination with interventions regarding household cleaning.^48^

BLLs observed in the district of Goslar exceeded those reported in another former mining area in Germany. In Euskirchen, where mining activities ceased in 1947, 18% of children exceeded the German RVs, with a median BLL of 10 µg/L in 2021 (see 'Euskirchen (2021)' in Table 4).^26^ It is important to note that the German RVs used at the time of this study were 15 µg/L for girls and 20 µg/L for boys.^49^ Applying these RVs to the present study, 65% of the children would exceed the respective German RVs, highlighting an even greater contrast between the two regions. This occurs despite their shared historical mining background and the fact that median soil lead concentrations in Euskirchen are substantially higher (approximately 4,000 mg/kg) than in Goslar (approximately 1,500 mg/kg). Another example is Kabwe, Zambia – one of the most heavily contaminated sites globally, with median soil lead concentrations around 6,000 mg/kg – where more than 95% of children had BLLs exceeding 100 µg/L in 2006.^50^

**Table 4.** Blood lead levels reported in different studies over time

Study (year)		Percentile			Mean	
		50	95	98	AM	GM
	N	µg/L				
Oker (1980)^38^	71	228	–	–	–	–
Hettstedt (1992–1993)^39^	527	38.0	39.5	–	–	38.0
GerES V (2014–2017)^28^	720	9.4	19.9	24.0	10.6	9.5
Oker and Harlingerode (2021)^42^	75	16.9	37.0	53.3	18,8	16.9
Euskirchen (2021)^26^	182	10.2	28.5	–	12.1	–
**Present study (2023–2024**)	**310**	**20.4**	**79.5**	**130.5**	**29.7**	**22.7**

AM = Arithmetic mean; GM = Geometric mean.

The differences in BLLs observed across different studies and regions suggest that lead exposure results not only from soil lead contamination but is influenced by other determinants, such as air quality, season, sex, age, SES and the intake of contaminated food.^51^

Median BLLs in children from this study were about 10% of 1980 levels (see 'Oker (1980)' in Table 4),^38^ reflecting the historical global decline following regulatory measures, such as the phase-out of leaded gasoline worldwide, which resulted in improved air quality.^7,52^

Seasonal variation in BLLs is well-documented,^26,39,53–55^ reflecting recent exposure due to lead’s biological half-life (~30 days).^48,56^ Similar to previous studies, we observed peak BLLs in summer, likely due to increased outdoor activity and greater contact with contaminated soil and dust in dry, warm conditions.^26,39,53–55^ Consistent with previous research,^26,27,28,39,42,57^ our study found higher BLLs in boys. This difference is likely due to boys’ larger erythrocyte fraction – since about 90% of blood lead binds to erythrocyte membranes^56^ – and typically more active outdoor behavior, which may increase exposure.^58^

Although younger children are generally more vulnerable to lead exposure,^7,26,28,39,42^ our study observed slightly higher BLLs in 6–7-year-olds than in 5-year-olds. Similar age-related patterns have been reported in other studies,^57,59–61^ suggesting that BLLs may initially rise with age before declining again around the age of school entry.^57^ This may also help to explain the lower BLLs found in the 2021 study conducted in Oker and Harlingerode in the district of Goslar (see 'Oker and Harlingerode (2021)' in Table 4),^42^ in which the participants were older (6–10 years of age) than those in the present study. Age-related differences may reflect changes in hygiene and play behavior with age. While frequent hand-to-mouth contact is common among younger children and recognized as a lead pathway,^26,62^ this was not significantly associated with BLLs in our 5–7-year-old cohort. Instead, handwashing after outdoor play and before eating – behaviors that tend to evolve with increasing age^63^ – were associated with lower BLLs. Our findings suggest that poor hygiene following environmental exposure may play a more important role than direct hand-to-mouth contact in 5–7-year-olds.

Some studies^7,27,28,31,39,55,64,65^ have reported that lower SES is associated with higher BLLs, although this difference has decreased in recent decades. SES likely influences lead exposure through factors such as environmental exposure (e.g., housing quality, secondhand smoke) and preventive behaviors. In our study, no statistically significant association between SES and BLLs was observed, which might be partly explained by the low participation rate of socioeconomically disadvantaged families in our study and the overall high living standards in high-income countries such as Germany. Consistent with our findings, a recent meta-analysis confirmed that exposure to secondhand tobacco smoke is associated with elevated BLLs in children across multiple studies.^24^ Plants grown in lead-contaminated soil can accumulate lead from the adherence of dust and translocation into the plant tissue.^8,66,67^ In Euskirchen, a former mining area in Germany with heavily contaminated soils, children who consumed homegrown food had significantly higher BLLs,^26^ which highlights the role of environmental background contamination. However, no such association was found in our study or in a similar study from Hettstedt, another former German mining region.^39^ In our analyses, variables such as cultivation method (raised beds vs. open ground) and hygiene practices (washing or peeling of food) had no effect on BLLs. A possible explanation for this discrepancy is the higher lead concentrations in soils in Euskirchen compared with Goslar,^26^ or the limited reliability of self-reported information collected via the interview in the present study. Our findings suggest that, under the conditions in Goslar, homegrown and foraged food was not a major exposure source.

Also, consumption of tap water from old lead pipes has been identified as a potential source of lead exposure in other studies in Germany.^68,69^ Nevertheless, in Hettstedt, a former mining area close to Goslar, lead in drinking water (GM 0.5 µg/L) was not statistically significantly associated with BLL.^70^ Tap water had already been excluded as a relevant exposure pathway in a previous investigation conducted in Goslar in 2021, with only 1 out of 29 samples exceeding the German reference value for lead in tap water (10 µg/L).^42^

An overview of the BLLs observed in the studies mentioned above is shown in Table 4.

### Strengths and limitations

Sensitivity analyses confirmed all associations reported, supporting the robustness of the results.

Children were recruited via letters sent to guardians alongside the official invitation to the compulsory school entry health check, ensuring broad reach across the preschool population in Goslar. The overall response rate was relatively high at 30%, exceeding that of previous German studies on children’s lead exposure – specifically, 23% in Oker and Harlingerode and 18% in Euskirchen, both conducted in 2021 and potentially affected by the SARS-CoV-2 pandemic.^26,42^

A limitation of the present study was the exclusion of 6% of participants due to incomplete data. Since individual BLLs were unknown at the time of recruitment, selection bias based on results seems unlikely. However, based on sociodemographic comparisons with official statistics,^71^ families with higher SES were likely overrepresented – a common pattern in health-related surveys.^72^ Nevertheless, the geographical distribution of participants’ residences closely mirrored that of all children invited to the school entry check, suggesting representativeness in terms of spatial lead exposure.

Capillary blood sampling, which is increasingly used in epidemiological studies due to its higher acceptability among children,^47^ was applied in this study and has been validated in a previous study by Strieker et al.^42^ (see supplementary information). In our study, only 5 samples (1.4%) exceeded the 20% RSD threshold between duplicates, suggesting potential imprecision. However, excluding these samples did not alter our results (Supplementary Table B3; https://links.lww.com/EE/A408). As the levels of quantifiable Pb in the tested blanks were relatively low, their influence on the overall results was considered negligible.

Outdoor activity is a major exposure factor for lead in former mining areas due to contact with contaminated soil and dust.^26,63,73^ However, in our study, no association was found between outdoor time and BLLs – likely due to imprecise exposure measurement, as outdoor time was only recorded in broad categories, limiting differentiation by location or duration, and the limited reliability of self-reported information. Therefore, a time-weighted aggregate soil lead exposure that might have provided a more accurate assessment of the relationship between soil lead contamination and BLLs could not be calculated.

Other potential exposure pathways for lead not assessed in this study include the child’s and guardian’s place of birth, housing characteristics (e.g., year of construction), residential setting (urban vs. rural, traffic exposure), lead-contaminated house dust, cleaning habits (e.g., frequency of cleaning, wearing outdoor shoes in the house), and parental occupations involving lead.^8,27,57,74^ These variables were excluded to shorten interview duration and maintain a high response rate. However, their exclusion represents an important limitation. Not accounting for these factors may have led to some confounding and could partly weaken or exaggerate the relationship between soil contamination and BLLs observed in our study.

## Conclusions

The present study demonstrates that children in the district of Goslar are more highly exposed to lead than the general population. The findings highlight the significant impact of lead soil contamination, particularly in residential areas, on children’s elevated BLLs.

Further preventive efforts and targeted interventions should be considered to reduce lead exposure. It is important to note that the issue of lead soil contamination is not limited to the district of Goslar. Regions worldwide with similar levels of soil lead contamination should also be included in public health interventions to protect children’s health.

## Conflict of interest statement

The authors declare that they have no conflicts of interest with regard to the content of this report.

## Supplementary Material


