# Supplementary material for: Blood lead levels in children and soil lead contamination in a former mining area in Germany
Source: Environ Epidemiol. 2026 Feb 9;10(2):e459. doi: 10.1097/EE9.0000000000000459 (PMC12889315; doi:10.1097/EE9.0000000000000459)
Supplement: Supplementary file 1 [file ee9-10-e459-s001.pdf]

Supplementary

Supplementary Table A1. Description of soil lead contamination in residential areas

Soil lead contamination	N	Minimum	10. Percentile	50. Percentile	90. Percentile	Maximum
Very high	456	14	412	1,523	6,827	30,189
High	150	14	177	406	851	3,274
Moderate	242	35	116	249	569	3,752
Low	174	21	52	97	193	4,000

Data from: District of Goslar. Goslar District Department of Construction and Environment. Regulation on the ‘Harz Land Use Planning Area in the District of Goslar’ (BPG-VO) (In German). 2023

Supplementary Table A2. P-values from pairwise comparisons (Mann–Whitney U test) of variables with more than two categories, with blood lead levels as continuous outcome

**Socioeconomic status**	Low	Moderate	High	–
Low	–	–	–	–
Moderate	0.22	–	–	–
High	0.32	0.64	–	–
**Season of blood sampling**	Winter	Autumn	Spring	Summer
Winter	–	–	–	–
Autumn	**< 0.01**	–	–	–
Spring	**< 0.01**	0.47	–	–
Summer	**< 0.01**	0.02	0.11	–
**Soil lead contamination at residence**	Low	Moderate	High	Very high
Low	–	–	–	–
Moderate	0.89	–	–	–
High	0.34	0.26	–	–
Very high	**0.02**	**0.01**	0.32	–

Socioeconomic status: low: all other, moderate: ≥ 1 guardian with intermediate school degree/vocational training, high: ≥ 1 guardian with university degree; Lead in soil (mg/kg): low < 200 mg/kg, moderate = 200−400 mg/kg, high = 400−1,000 mg/kg, very high > 1,000 mg/kg; statistically significant values ($p < 0.05$) in bold

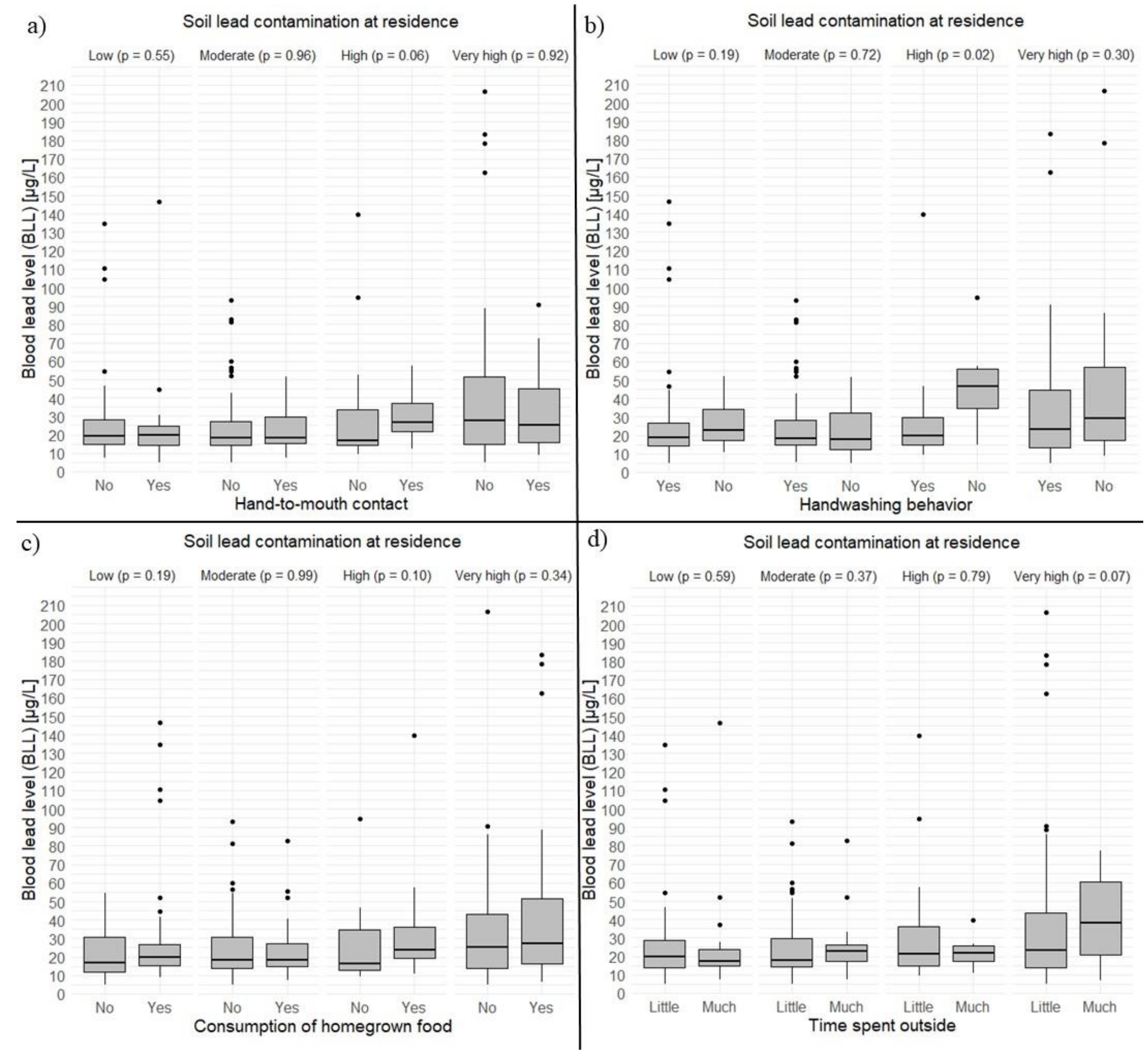

p-values for continuous BLLs; Kruskal-Wallis test; Lead in soil (mg/kg): low < 200 mg/kg, moderate = 200−400 mg/kg, high = 400−1,000 mg/kg, very high > 1,000 mg/kg; d) little: ≤ 15 h/week, much: > 15 h/week

Supplementary Figure A1. Hand-to-mouth contact, handwashing behavior, consumption of homegrown food, time spent outside stratified by soil lead contamination at residence

Supplementary Table A3. Description and blood lead levels by soil lead contamination at different locations

		Percentile					Mean [a]		Reference value [b]	
		25	50	75	95	98	AM	GM	GER	U.S.
	N	µg/L							N (%)	
Soil lead contamination at kindergarten (**p < 0.01**)										
Low	69	14.3	17.8	26.1	44.8	53.6	22.1	19.2	29 (42.0)	7 (10.1)
Moderate	92	14.3	18.9	30.1	70.2	137.0	28.1	21.2	41 (44.6)	16 (17.4)
High	42	15.2	19.5	33.3	62.9	102.9	27.9	21.7	20 (47.6)	9 (21.4)
Very high	77	16.7	29.3	51.5	91.2	172.2	40.5	29.9	51 (66.2)	37 (48.1)
Soil lead contamination at playgrounds or sports fields (p = 0.06)										
Low	61	14.3	17.7	26.0	44.7	52.4	22.1	19.2	25 (41.0)	6 (9.8)
Moderate	65	14.3	18.2	30.6	55.2	83.0	25.4	20.3	28 (43.1)	13 (20.0)
High	20	16.2	20.4	32.4	89.4	113.7	32.0	24.1	17 (56.7)	6 (20.0)
Very high	82	15.6	25.2	42.6	90.7	170.1	37.3	26.3	48 (58.5)	29 (35.4)
Soil lead contamination at forests or agricultural areas (p = 0.08)										
Low	50	14.4	19.0	32.5	53.4	58.0	26.3	21.6	23 (46.0)	9 (18.0)
Moderate	60	14.8	19.5	27.0	58.9	102.4	25.8	21.2	27 (45.0)	9 (15.0)
High	48	14.8	19.6	30.5	90.6	178.5	30.3	21.8	24 (50.0)	8 (16.7)
Very high	53	16.3	26.9	43.2	82.7	137.8	36.2	27.9	33 (62.3)	20 (37.7)
Soil lead contamination at private garden (**p = 0.01**)										
Low	80	14.8	19.8	27.6	56.8	120.6	27.0	21.6	39 (48.8)	13 (16.2)
Moderate	70	14.9	18.6	29.6	58.2	82.2	25.6	20.9	32 (45.7)	13 (18.6)
High	32	15.0	22.3	37.1	74.2	111.9	31.2	25.1	19 (59.4)	9 (28.1)
Very high	75	16.5	31.7	53.1	112.2	180.7	42.3	30.3	49 (65.3)	34 (45.3)
Soil lead contamination at friends'/relative's gardens (p = 0.48)										
Low	53	14.9	18.7	24.7	47.6	102.4	24.3	19.9	22 (41.5)	6 (11.3)
Moderate	37	13.9	18.3	29.1	42.6	53.9	22.8	19.9	16 (43.2)	4 (10.8)
High	16	14.9	20.3	39.4	98.6	105.6	33.9	25.5	9 (56.2)	5 (31.2)
Very high	48	15.3	21.5	40.5	73.9	184.4	34.8	24.7	26 (54.2)	13 (27.1)
Soil lead contamination at waterside locations (p = 0.27)										
Low	21	14.4	18.6	27.5	56.2	85.2	25.3	20.9	9 (42.9)	3 (14.3)
Moderate	15	12.5	17.6	30.6	56.0	56.8	23.4	18.6	6 (40.0)	3 (20.0)
High	40	15.9	21.3	34.4	96.8	137.5	31.8	24.2	20 (50.0)	9 (22.5)
Very high	67	15.2	26.8	42.6	68.9	81.0	31.2	25.4	44 (65.7)	22 (32.8)

[a] AM = Arithmetic mean, GM = Geometric mean; [b] German (GER) reference values: 19 µg/L for girls, 22 µg/L for boys; U.S. reference value: 35 µg/L for both sexes; lead in soil (mg/kg): low < 200 mg/kg, moderate = 200−400 mg/kg, high = 400−1,000 mg/kg, very high > 1,000 mg/kg; p-values for continuous BLLs; Kruskal-Wallis test; statistically significant values ($p < 0.05$) in bold

Supplementary Table B1. Group comparison across all variables with blood lead levels as binary outcome variables

		Reference value [a]	
	N	GER	U.S.
Total	310	175 (50.6)	75 (24.2)
Sex		**$p = 0.02$** [b]	**$p < 0.01$** [b]
Female	136	58 (42.6)	19 (14.0)
Male	174	99 (56.9)	56 (32.2)
Age		$p = 0.08$ [b]	$p = 0.05$ [b]
5 years	201	94 (46.8)	41 (20.4)
6−7 years	109	63 (57.8)	34 (31.2)
Socioeconomic Status [d]		$p = 0.35$ [c]	$p = 0.62$ [c]
Low	20	7 (35.0)	4 (20.0)
Moderate	141	74 (52.5)	37 (26.2)
High	139	69 (49.6)	30 (21.6)
Exposure to second-hand tobacco smoke [e]		$p = 0.33$ [b]	$p = 0.05$ [b]
No	234	114 (48.7)	49 (20.9)
Yes	73	41 (56.2)	24 (32.9)
Hand-to-mouth contact [f]		$p = 0.58$ [b]	$p = 0.46$ [b]
No	210	104 (49.5)	54 (25.7)
Yes	95	51 (53.7)	20 (21.1)
Handwashing behavior [e]		$p = 0.06$ [b]	$p = 0.12$ [b]
No	65	40 (61.5)	21 (32.3)
Yes	242	115 (47.5)	53 (21.9)
Consumption of homegrown food [g]		$p = 0.09$ [b]	$p = 0.45$ [b]
No	126	56 (44.4)	34 (27.0)
Yes	182	100 (54.9)	41 (22.5)
Consumption of food from the forest [g]		$p = 0.44$ [c]	$p = 0.18$ [c]
No	279	139 (49.8)	65 (23.3)
Yes	29	17 (58.6)	10 (34.5)
Soil lead contamination [h] at residence		$p = 0.08$ [b]	**$p < 0.01$** [b]
Low	85	39 (45.9)	13 (15.3)
Moderate	91	39 (42.9)	14 (15.4)
High	39	22 (56.4)	10 (25.6)
Very high	95	57 (60.0)	38 (40.0)
Time spent outside [i]		$p = 0.50$ [b]	$p = 0.92$ [b]
Little	237	117 (49.4)	57 (24.1)
Much	73	40 (54.8)	18 (24.7)
Season of blood sampling		**$p < 0.01$** [b]	**$p < 0.01$** [b]
Autumn	81	46 (56.8)	15 (18.5)
Winter	97	34 (35.1)	11 (11.3)
Spring	79	44 (55.7)	27 (34.2)
Summer	53	33 (62.3)	22 (41.5)

Table continuous on next page.

		Reference value [a]	
	N	GER	U.S.
Soil lead contamination [h] at kindergarten		**p = 0.01** [c]	**p < 0.01** [c]
Low	69	29 (42.0)	7 (10.1)
Moderate	92	41 (44.6)	16 (17.4)
High	42	20 (47.6)	9 (21.4)
Very high	77	51 (66.2)	37 (48.1)
Soil lead contamination [h] at playgrounds or sports fields		p = 0.11 [c]	**p < 0.01** [c]
Low	61	25 (41.0)	6 (9.8)
Moderate	65	28 (43.1)	13 (20.0)
High	20	17 (56.7)	6 (20.0)
Very high	82	48 (58.5)	29 (35.4)
Soil lead contamination [h] at forests or agricultural areas		p = 0.26 [c]	**p = 0.02** [c]
Low	50	23 (46.0)	9 (18.0)
Moderate	60	27 (45.0)	9 (15.0)
High	48	24 (50.0)	8 (16.7)
Very high	53	33 (62.3)	20 (37.7)
Soil lead contamination [h] at private garden		**p = 0.01** [c]	**p < 0.01** [c]
Low	80	39 (48.8)	13 (16.2)
Moderate	70	32 (45.7)	13 (18.6)
High	32	19 (59.4)	9 (28.1)
Very high	75	49 (65.3)	34 (45.3)
Soil lead contamination [h] at friends'/relative's gardens		p = 0.50 [c]	p = 0.59 [c]
Low	53	22 (41.5)	6 (11.3)
Moderate	37	16 (43.2)	4 (10.8)
High	16	9 (56.2)	5 (31.2)
Very high	48	26 (54.2)	13 (27.1)
Soil lead contamination [h] at waterside locations		p = 0.10 [c]	p = 0.35 [c]
Low	21	9 (42.9)	3 (14.3)
Moderate	15	6 (40.0)	3 (20.0)
High	40	20 (50.0)	9 (22.5)
Very high	67	44 (65.7)	22 (32.8)

[a] German (GER) reference values: 19 µg/L for girls, 22 µg/L for boys; U.S. reference value: 35 µg/L for both sexes; [b] p-values for binary BLLs; Chi-square test; [c] p-values for binary BLLs; Fisher's exact test; statistically significant values ($p < 0.05$) in bold; [d] N = 10 missings; [e] N = 3 missings; [f] N = 5 missings; [g] N = 2 missings; [h] lead in soil (mg/kg): low < 200 mg/kg, moderate = 200−400 mg/kg, high = 400−1,000 mg/kg, very high > 1,000 mg/kg; [i] little: ≤ 15 h/week, much: > 15 h/week

Supplementary Table B2. Logistic regression model of blood lead levels by residential soil lead contamination (N = 304)

	**German reference values**				**U.S. reference value**			
	**Unadjusted**		**Adjusted**		**Unadjusted**		**Adjusted**	
	**OR**	**95% CI**	**OR**	**95% CI**	**OR**	**95% CI**	**OR**	**95% CI**
Intercept	0.87	0.56–1.33	**0.11**	**0.05–0.27**	**0.18**	**0.10–0.32**	**0.01**	**0.01–0.02**
Soil lead contamination at residence								
Low	1	–	1	–	1	–	1	–
Moderate	0.86	0.47–1.57	0.98	0.52–1.88	1.02	0.45–2.34	1.17	0.48–2.89
High	1.49	0.70–3.24	2.21	0.96–5.20	**1.88**	**0.73–4.77**	**3.29**	**1.12–9.71**
Very high	1.64	0.91–3.00	1.92	1.00–3.73	**3.35**	**1.66–7.13**	**5.19**	**2.28–12.46**
Sex								
Female	–	–	1	–	–	–	1	–
Male	–	–	**2.16**	**1.32–3.57**	–	–	**4.61**	**1.26–9.49**
Age								
5 years	–	–	1	–	–	–	1	–
6–7 years	–	–	**2.47**	**1.28–4.90**	–	–	**2.98**	**1.24–7.45**
Second-hand tobacco smoke								
No	–	–	1	–	–	–	1	–
Yes	–	–	1.27	0.72–2.24	–	–	**2.21**	**1.11–4.38**
Handwashing behavior								
Yes	–	–	1	–	–	–	1	–
No	–	–	1.76	0.96–3.30	–	–	1.65	0.79–3.38
Season of blood sampling								
Winter	–	–	1	–	–	–	1	–
Autumn	–	–	**4.37**	**2.10–9.49**	–	–	**4.61**	**1.58–14.41**
Spring	–	–	**4.51**	**2.18–9.73**	–	–	**14.43**	**5.17–45.08**
Summer	–	–	**2.82**	**1.30–6.21**	–	–	**7.35**	**2.78–20.76**

German reference values: 19 µg/L for girls, 22 µg/L for boys; U.S. reference value: 35 µg/L for both sexes; OR = Odds Ratio; 95% CI = 95% confidence interval; statistically significant values in bold; lead in soil (mg/kg): low < 200 mg/kg, moderate = 200–400 mg/kg, high = 400–1,000 mg/kg, very high > 1,000 mg/kg; adjusted R^2 for adjusted model (GER/U.S.) = 0.17/0.33

Supplementary Table B3. Unadjusted and adjusted regression models with blood lead levels as a continuous outcome for factors potentially associated with elevated blood lead levels excluding children with ammunition exposure or RSD > 20% (N = 292)

	**Unadjusted**		**Adjusted**	
	**Coefficient (β)**	**95% CI**	**Coefficient (β)**	**95% CI**
Intercept	**20.87**	**17.90–24.34**	**10.81**	**8.55–13.68**
Soil lead contamination at residence				
Low	1	—	1	—
Moderate	0.96	0.78–1.18	1.00	0.82–1.21
High	1.12	0.86–1.46	1.26	0.98–1.62
Very high	**1.27**	**1.03–1.57**	**1.30**	**1.07–1.58**
Sex				
Female	—	—	1	—
Male	—	—	**1.37**	**1.19–1.59**
Age				
5 years	—	—	1	—
6−7 years	—	—	1.15	0.95–1.40
Second-hand tobacco smoke				
No	—	—	1	–
Yes	—	—	1.17	0.98–1.39
Handwashing behavior				
Yes	—	—	1	—
No	—	—	1.20	1.00–1.44
Season of blood sampling				
Winter	—	—	1	—
Autumn	—	—	**1.41**	**1.14–1.74**
Spring	—	—	**1.69**	**1.37–2.09**
Summer	—	—	**1.79**	**1.42–2.25**

RSD = Relative Standard Deviation; Coefficient β = Exponentiated regression coefficient (β); BLLs were log-transformed; 95% CI = 95% confidence interval; statistically significant values in bold; lead in soil (mg/kg): low < 200 mg/kg, moderate = 200−400 mg/kg, high = 400−1,000 mg/kg, very high > 1,000 mg/kg; adjusted R^2 for adjusted model = 0.18
